# Achalasia and Esophageal Motility Disorders in the Bariatric Surgery Population: A Retrospective Descriptive Series

**DOI:** 10.1007/s11695-026-08510-x

**Published:** 2026-03-18

**Authors:** Pattharasai Kachornvitaya, Melissa V. Wills, Baraa K. Mohamed, Valentin Mocanu, Juan S. Barajas-Gamboa, Xinlei Zhu, Yung Lee, Ricard Corcelles, Andrew T. Strong, Suthep Udomsawaengsup, Salvador Navarrete, Jerry Dang, Matthew Kroh

**Affiliations:** 1 Digestive Disease Institute, Cleveland Clinic, Cleveland, United States; 2 Cleveland Clinic Lerner College of Medicine, Cleveland, United States; 3 Department of Surgery, Northeast Georgia Medical Center, Gainesville, United States; 4 Digestive Diseases Institute, Cleveland Clinic Abu Dhabi, Abu Dhabi, United Arab Emirates; 5 Department of Surgery, Faculty of Medicine, Chulalongkorn University, Bangkok, Thailand; 6 Treatment of Obesity and Metabolic Disease Research Unit, Faculty of Medicine, Chulalongkorn University, Bangkok, Thailand

**Keywords:** Achalasia, Bariatric surgery, Obesity, Esophageal motility disorders, POEM, Heller myotomy, Surgical algorithm

## Abstract

**Background:**

Achalasia and severe obesity represent a rare but clinically challenging intersection. In patients undergoing bariatric surgery, esophageal motility disorders (EMDs), including achalasia and related conditions, may be pre-existing, unmasked, or develop following surgical alteration of foregut anatomy. Optimal treatment sequencing in this population remains poorly defined.

**Objectives:**

To descriptively characterize clinical presentation, diagnostic features, and outcomes in patients treated for both severe obesity and EMDs, and to propose a preliminary treatment algorithm based on treatment sequencing.

**Methods:**

We conducted a retrospective descriptive case series of patients treated between 2008 and 2023 at a single academic center. Among 7,054 patients who underwent primary sleeve gastrectomy (SG) or Roux-en-Y gastric bypass (RYGB), 17 patients also underwent procedures for achalasia and EMDs were included. Patients were categorized by treatment sequence: achalasia treatment first (AF), bariatric surgery first (BF), or concurrent treatment.

**Results:**

The cohort had a mean age of 56.9 years and was predominantly female (70.6%). Type II achalasia was most common (41.2%). Patients treated for achalasia before or concurrently with bariatric surgery (*n* = 9) demonstrated generally favorable outcomes, with 12-month percentage total weight loss (%TWL) ranging from 20.2% to 49.6% and sustained symptom control in 66.7%. In contrast, patients treated for achalasia or EMDs after bariatric surgery (*n* = 8) experienced more heterogeneous clinical courses, with frequent need for additional interventions and variable symptom control, particularly following SG. Esophageal dysfunction occurred earlier after SG than after RYGB, and three of four SG patients (75%) required conversion to RYGB.

**Conclusions:**

In this small descriptive series, treatment sequencing appeared to influence clinical outcomes in patients with severe obesity and achalasia or EMDs. Given the diagnostic complexity and heterogeneity of this population, these findings should be interpreted as hypothesis-generating. Larger, multicenter studies with standardized diagnostic criteria are needed to inform evidence-based management strategies.

**Supplementary Information:**

The online version contains supplementary material available at 10.1007/s11695-026-08510-x.

## Highlights

Achalasia and esophageal motility disorders occurred in 0.24% of bariatric patients.

Treatment before or concurrent with bariatric surgery showed more favorable courses.

Esophageal dysfunction was identified earlier after sleeve gastrectomy than RYGB.

Many sleeve gastrectomy patients required conversion to Roux-en-Y gastric bypass.

Concurrent achalasia and bariatric surgery was feasible in a selected patient.

**Supplementary Information:**

The online version contains supplementary material available at 10.1007/s11695-026-08510-x.

## Introduction

Obesity is a global health concern linked to significant comorbidities, including gastroesophageal reflux disease (GERD) and esophageal motility disorders (EMDs) such as achalasia, esophagogastric junction outflow obstruction (EGJOO), and ineffective esophageal motility (IEM) [1–3]. EMDs are increasingly identified during preoperative evaluations for bariatric surgery, particularly in patients presenting with dysphagia [3]. Bariatric procedures, notably Roux-en-Y gastric bypass (RYGB) and sleeve gastrectomy (SG), yield substantial weight loss and metabolic improvements but also alter gastrointestinal physiology, potentially impacting esophageal motility [1, 4].

Achalasia, a rare EMD with an incidence of ~ 1 per 100,000 annually, involves impaired lower esophageal sphincter (LES) relaxation and loss of peristalsis due to degeneration of the myenteric plexus [5–7]. Standard treatments such as pneumatic dilation, Heller myotomy (HM), peroral endoscopic myotomy (POEM), and botulinum toxin vary in efficacy and durability [8]. Obesity has been associated with a high prevalence of esophageal dysmotility, with achalasia reported in up to 1% of individuals with severe obesity [9, 10], and approximately 70% of achalasia patients meeting criteria for overweight or obesity [11, 12].

The coexistence of obesity and achalasia and their spectrum present diagnostic and therapeutic challenges, with overlapping symptoms complicating diagnosis and management. Bariatric surgery may be pre-existing, unmasked, or develop following surgical alteration of foregut anatomy, and contributing to post-obesity esophageal dysfunction (POSED) in up to 40% of cases [9, 13]. Moreover, anatomical changes may induce de novo achalasia or EMDs [9, 14]. Despite increasing clinical relevance, data on patients undergoing both achalasia or EMDs treatment and bariatric surgery remain sparse, with most reports limited to case studies.

This retrospective descriptive case series examines clinical outcomes in patients undergoing achalasia or EMDs treatment and bariatric surgery, with a focus on treatment sequencing. By stratifying patients according to the timing of achalasia or EMDs diagnosis and intervention relative to bariatric surgery, before, after, or concurrent, we descriptively assess symptom response, weight loss, complications, and need for reintervention in this rare and complex population. The findings are intended to be hypothesis-generating and to inform a preliminary, experience-based treatment framework.

## Methods

### Study Design and Setting

This study is a retrospective descriptive series from a prospective database conducted at a single academic institution between 2008 and 2023. The study was designed as a descriptive case series to characterize clinical outcomes in the rare population of patients requiring treatment for both achalasia and severe obesity. Institutional Review Board (IRB) approval was obtained prior to data collection. Given the rarity of this clinical intersection and the descriptive nature of the study objectives, no sample size calculations were performed.

### Patient Identification and Selection

A systematic approach was used to identify all eligible patients during the study period. Electronic medical records were reviewed to identify patients who had undergone any form of procedure for achalasia in the operating room or endoscopy suite, including POEM, Heller myotomy, pneumatic dilation, and Botulinum toxin injection using CPT codes 43,499, 43,279, 43,450, and 43,201, respectively. Simultaneously, patients with a history of SG or RYGB, using CPT codes 43,775 and 43,644, respectively, were identified. Only patients who had undergone procedures for both severe obesity and achalasia or EMDs were included.

### Study Population

Patients were classified according to the timing of achalasia or achalasia-spectrum disorder diagnosis and treatment relative to bariatric surgery. Patients who received treatment for achalasia prior to or concomitant with bariatric surgery were categorized as the achalasia-first (AF) group. Patients diagnosed and treated for achalasia after bariatric surgery were categorized as the bariatric-first (BF) group.

### Data Collection

Data collected included demographic information, initial presenting symptoms, diagnostic study included upper endoscopy, high-resolution manometry (HRM), and timed barium esophagogram when clinically indicated, EMDs subtype, treatment modality, interval between EMDs treatment and bariatric surgery, type of bariatric procedure performed, percentage of total weight loss (%TWL) at one year and at the last available follow-up, bariatric surgery-related complications, response to achalasia treatment, and any additional interventions required. Data were extracted from electronic medical records using a standardized data collection form. Missing data were noted and reported for each variable.

HRM findings were interpreted in conjunction with clinical presentation and prior foregut anatomy. Diagnoses were categorized according to the Chicago Classification version 4.0 [15] when complete data were available. Patients with classic achalasia subtypes demonstrated impaired lower esophageal sphincter relaxation and absent peristalsis. Patients with EGJOO or preserved peristalsis were included only when clinical features, imaging, and endoscopic evaluation supported an EMDs, including POSED. In cases with incomplete or unavailable manometric data, diagnoses were based on integrated clinical documentation. To avoid diagnostic overstatement, these cases are referred to as EMDs rather than classic achalasia.

### Outcome Definitions

Primary outcomes included symptom resolution, weight loss metrics, and bariatric procedural complications. Secondary outcomes included the need for reinterventions and long-term follow-up status. Symptom resolution was defined as patient-reported improvement in dysphagia and related symptoms as documented in clinical notes.

Standardized symptom scores such as the Eckardt score were not used because the inclusion of weight loss may confound outcomes in bariatric populations.

### Operative Strategy and Perioperative Considerations

Surgical strategies were individualized based on prior foregut anatomy and disease severity. In patients undergoing Heller myotomy prior to bariatric surgery, a partial fundoplication was performed at the time of myotomy and subsequently taken down during RYGB to facilitate gastric pouch creation. Bilateral truncal vagotomy was not routinely performed in patients undergoing gastric bypass. In patients with prior sleeve gastrectomy, no concomitant anti-reflux procedure was performed at the time of myotomy; postoperative management consisted of acid suppression therapy.

Selection of achalasia treatment was individualized based on achalasia subtype, prior foregut anatomy, symptom severity, and multidisciplinary discussion involving foregut and bariatric surgeons. POEM and Heller myotomy were favored for definitive therapy, while endoscopic therapies were generally used in high-risk patients or as temporizing measures.

### Statistical Analysis

Descriptive statistics were employed to summarize patient demographics and clinical outcomes. Categorical variables were reported as frequencies and percentages. Continuous variables were presented as means with standard deviations or medians with interquartile ranges (IQR), depending on data distribution. All statistical analyses were conducted using R version 4.3.2 (R Core Team, 2024) within the RStudio environment (RStudio Team, 2023). Given the small sample size and descriptive nature of the study, no inferential statistical tests were conducted. No formal comparisons between groups were performed due to the limited sample size and the descriptive study objectives.

## Results

### Patient Demographics and Baseline Characteristics

Of the 7,054 patients who underwent primary SG or RYGB during the study period, 17 patients were included. This corresponds to a prevalence of 0.24% within our bariatric surgery population, with a mean patient age of 56.9 ± 7.6 years. The majority were female (70.6%) and White (70.6%). The AF group and BF group were similar in age and race; however, more females were in the AF group (77.8% vs. 62.5%). The AF group had a higher mean BMI (48.3 ± 10.3 vs. 41 ± 7.7 kg/m²). Common comorbidities included obstructive sleep apnea (76.5%) and GERD (76.5%), with higher OSA prevalence in the BF group (87.5%). Diabetes was more prevalent in the AF group (33.3% vs. 12.5%). Full details in Table 1.

**Table 1:** Baseline characteristics and treatment details of patients

Patient characteristics	Total(*n* = 17)	AFG(*n* = 9)	BFG(*n* = 8)
Age (years)	56.9 ± 7.6	56.4 ± 7.2	57.5 ± 8.4
Female (n, %)	12 (70.6)	7 (77.8)	5 (62.5)
Race (n, %)			
White	12 (70.6)	7 (77.8)	6 (75)
Black or African American	5 (29.4)	2 (22.2)	2 (25)
Body mass index (kg/m2)	44.6 ± 9.6	48.3 ± 10.3	41 ± 7.7
**Comorbidities** (n, %)			
Diabetes	3 (17.6)	3 (33.3)	1 (12.5)
Hypertension	7 (41.2)	3 (33.3)	4 (50)
Dyslipidemia	3 (17.6)	1 (11.1)	2 (25)
Chronic obstructive pulmonary disease	3 (17.6)	2 (22.2)	1 (12.5)
Obstructive sleep apnea	13 (76.5)	6 (66.7)	7 (87.5)
Gastroesophageal reflux disease	13 (76.5)	7 (77.8)	6 (75)
Anxiety/Depression	2 (11.8)	1 (11.1)	1 (12.5)
**EMDs treatment details (n**,**%)**			
Initial treatment			
Botulinum toxin injection	4 (28.6)	2 (22.3)	2 (25)
POEM	5 (35.7)	3 (33.3)	2 (25)
Heller myotomy	3 (21.4)	3 (33.3)	-
Heller myotomy + RYGB	2 (14.3)	1 (11.1)	2 (25)
Others			2 (25)
Additional treatment			
Botulinum toxin injection	3 (33.3)	3 (42.8)	-
Pneumatic dilation	3 (33.3)	2 (28.6)	1 (25)
POEM	1 (11.2)	-	1 (25)
Heller myotomy	2 (22.2)	2 (28.6)	2 (50)
**Bariatric surgery (n**,** %)**			
SG	4 (23.5)	0 (0)	4 (50)
RYGB	12 (76.5)	8 (100)	4 (50)
SG to RYGB	3 (75)	-	3 (75)
Interval period between treatments, years (median, IQR)			
EMDs treatment to bariatric surgery	-	2.3 (1.1, 4.3)	-
Bariatric surgery to EMDs treatment		-	7.3 (5.4, 19.0)
SG		-	6.0 (5.1, 7.3)
RYGB		-	19.8 (10.2, 25.1)
Length of stay, days (median, IQR)			
After achalasia treatment	2 (1.5, 3.5)	2 (1, 4)	3 (1, 3)
After bariatric surgery	3 (2, 4)	4 (2, 4)	3 (2.8, 3.3)
Concurrent treatment	-	2	-
Longest Follow-up, years (median, IQR)	10 (3.2, 19.3)	3.2 (2.5, 8.4)	13.5 (9.2, 24)

*AFG* Achalasia first group, *BFG* Bariatric first group, *EMDs* Esophageal motility disorders, *POEM* Peroral endoscopic myotomy, *RYGB* Roux-en-Y gastric bypass, *SD* Standard deviation, *IQR* Interquartile range

### Achalasia Presenting Symptoms and HRM

At presentation, the most common symptom was dysphagia to solids (82.4%), followed by regurgitation (58.8%), heartburn (52.9%), chest pain (47.1%), and dysphagia to liquids (41.2%) (Table S1).

HRM showed type II achalasia in 41.2%. Type I and III achalasia were each found in 5.9% of patients. EGJOO was seen in 23.5%, and one patient had findings consistent with POSED. In two patients, the achalasia subtype could not be specified; the diagnosis was based on clinical documentation (Table S2).

### Achalasia Treatment Modalities

POEM was the most common initial achalasia treatment, followed by botulinum toxin injection, HM, and combined HM with RYGB. AF group predominantly received POEM (37.5%) or HM (37.5%), while BF group had a more even distribution among POEM, botulinum toxin, and combined procedures (each 33.3%). Additional interventions, particularly botulinum toxin and pneumatic dilation, were more frequent in the AF group. HM was more often required in the BF group. Median hospital stay post-achalasia treatment was slightly longer in the BF group (3 days, IQR 1–3) compared to the AF group (2 days, IQR 1–4).

### Bariatric Surgery Characteristics

Patients in the AF group underwent RYGB exclusively (100%), while those in the BF group included both RYGB (50%) and SG (50%), with three out of four SG patients (75%) requiring conversion to RYGB. After bariatric surgery, AF group had a marginally longer median hospital stay (4 days, IQR 2–4) than BF group (3 days, IQR 2.8–3.3) (Table 1).

### Treatment Timing and Intervals

Patients were grouped by treatment sequence: AF group (*n* = 8, 47.1%), BF group (*n* = 8, 47.1%), and concurrent treatment (*n* = 1, 5.8%). In the AF group, the median interval between achalasia and bariatric surgery was 2.3 years (IQR 1.1–4.3); in the BF group, the median interval from bariatric surgery to achalasia diagnosis was 7.3 years (IQR 5.4–19). Among BF group, achalasia developed later after RYGB (19.8 years, IQR 10.2–25.1) than SG (6.0 years, IQR 5.1–7.3) (Table 1).

### Outcomes in the Achalasia First Group

Among eight AF group, initial treatments included POEM (*n* = 3), HM (*n* = 3), and botulinum toxin (*n* = 2). Two patients initially treated with botulinum toxin required definitive therapy with PD and HM. One patient who initially underwent HM required subsequent botulinum toxin injections and ultimately redo HM for recurrent dysphagia. One patient treated with POEM required adjunctive PD and botulinum toxin injections during follow-up after an intramural leak that was managed endoscopically. All patients later underwent RYGB. At 12 months, %TWL ranged from 20.2% to 33.9%, with one patient achieving 56.3% at 14 years. One patient developed a gastrojejunostomy stricture requiring dilation. Overall, this group had favorable long-term weight loss and symptom control (Table 2).

**Table 2:** Clinical outcomes in achalasia first (AF) or concurrent treatment group

No.	Type of Achalasia	Initial Treatment of Achalasia	Myotomy Length (cm)	Outcomes	Additional Treatment	Interval between two treatments (years)	Bariatric Surgery	%TWL at 12 months	Bariatric Complication	Additional Treatment (Outcomes)	%TWL at last visit*
1	II	POEM	9	Improved dysphagia	-	4.8	RYGB	26.3%	none	-	25.6% (2 years)
2	I	HM	4.5	Recurrent dysphagia	Botulinum toxin x2, Redo HM	4.2	RYGB	20.2%	none	-	18% (11 years)
3	III	POEM	N/A	Intramural leak (Stent)	Botulinum toxin, Pneumatic dilation	1.4	RYGB	N/A	none	-	19.9% (7 years)
4	I	Botulinum toxin	-	Partially improved dysphagia	Pneumatic dilation	9.0	RYGB	30.7%	none	Botulinum toxin (Improved)	56.3% (14 years)
5	EGJOO	Botulinum toxin	14	Partially improved dysphagia	HM	0.2	RYGB	28.3%	GJ stricture	Dilation (Improved)	N/A (3 year)
6	II	HM and epiphrenic diverticulectomy	6	Improved dysphagia	PEH repair at 8 months (mesh and gastropexy)	2.2	RYGB	30.3%	none	-	30.3% (1 year)
7	II	POEM	13	Improved dysphagia	-	0.3	RYGB	33.9%	none	-	33.9% (1 year)
8	Unknown	OHM	N/A	N/A	N/A	2.3	RYGB	N/A	none	-	8.3% (2 months)
9	4	HM^†^	10	Improved dysphagia	-		RYGB^†^	N/A	none	-	49.6% (1.3 years)

*TWL* Total weight loss, *POEM* Peroral endoscopic myotomy, *RYGB* Roux-en-Y gastric bypass, *HM* Heller myotomy, *EGJOO* Esophagogastric junction outflow obstruction, *GJ* Gastrojejunostomy, *PEH* Paraesophageal hernia, *OHM* Open Heller myotomy, *N/A* No available data, Unknown = no subtype specification

*Postoperative interval following bariatric surgery

^†^Simultaneous achalasia and bariatric surgery

One patient underwent simultaneous achalasia and bariatric surgery with a combined HM and RYGB. Postoperatively, the patient reported resolution of dysphagia and only mild reflux symptoms. At 1.3 years of follow-up, the %TWL was 49.6%.

### Outcomes in the Bariatric First Group

Eight patients underwent evaluation and management for achalasia or EMDs following bariatric surgery (four after RYGB or four after SG). Six patients underwent achalasia-directed interventions, while two were managed conservatively without myotomy. Weight loss data at 12 months were available for three sleeve gastrectomy patients, with %TWL of 22.9%, 26.2%, and 16.8%, respectively. One patient experienced subsequent weight recurrence, with %TWL declining to 19.7% at 8.3 years, whereas another demonstrated sustained weight loss of 29.9% at 5.7 years. Achalasia or EMDs treatments included POEM [3], HM [1], and HM with RYGB [2]. In some patients, endoscopic therapies such as botulinum toxin injection and pneumatic dilation were used as initial or temporizing measures before definitive treatment. Clinical outcomes varied, with recurrent dysphagia, reflux, and need for escalation to surgical intervention observed in several patients, reflecting the complexity of managing esophageal motility disorders in the post-bariatric setting (Table 3).

**Table 3:** Clinical outcomes in bariatric first (BF) group

No.	BS	%TWL at 12 months	Bariatric complication (Treatment)	%TWL prior to treatment	Interval between BS and EMDs (yrs)	Manometric Classification	Treatment	Myotomy Length (cm)	Outcomes	Additional Treatment (Outcomes)	%TWL at last visit*
9	RYGB	N/A	1. weight regain, GG fistula, marginal ulcer (GJ Revision, Proximal gastrectomy) 2. Internal hernia (Reoperation for mesenteric defect closure)	N/A	18.3	EGJOO	Botulinum toxin, Pneumatic dilation, HM	8.5	Recurrent dysphagia, GERD	Medical treatment (Improved)	N/A (23 years)
10	RYGB	N/A	-	39.10%	2	II	POEM	13	Recurrent dysphagia	HM (Improved)	11.6% (10 years)
11	SG	N/A	N/A	N/A	6.3	Unknown	HM + RYGB	14	GJ ulcer perforation, Dysphagia due to stenosis at hiatus	Distal esophagectomy with gastrectomy with esophagojejunostomy (Improved)	13.8% (6.9 years)
12	SG	22.9%	-	19.70%	8.3	II	HM + RYGB	8	-	-	27.6% (15.5 years)
13	RYGB	N/A	-	N/A	21.2	II	POEM	13	Reflux	N/A	7.7% (26.9 years)
14	SG	26.2%	EGJ stenosis (stent), Dysphagia	29.90%	5.7	II	Botulinum toxin, POEM	8	Severe GERD	Dilation, Conversion to RYGB (Improved)	26.2% (11.4 years)
15	RYGB	N/A	GG fistula	N/A	29	EGJOO	-	-	-	Excision GG fistula, GJ revision (Improved)	22.6% (36.5 years)
16	SG	16.8%	Sleeve stenosis (dilation) Hiatal hernia (repair)	20.4%	4.5	POSED	-	-	GERD	Medical treatment (Improved)	18% (5.8 years)

*BS* Bariatric surgery, *TWL* Total weight loss, *EMDs* Esophageal motility disorders, *EGJOO* Esophagogastric junction outflow obstruction, *POEM* Peroral endoscopic myotomy, *RYGB* Roux-en-Y gastric bypass, *SG* Sleeve gastrectomy, *HM* Heller myotomy, *GJ* Gastrojejunostomy, *SBO* Small bowel obstruction, *GERD* Gastroesophageal reflux disease, *GG* Gastro-gastric, *POSED* Post obesity surgery esophageal dysfunction, *N/A* No available data, Unknown = no subtype specification

*Postoperative interval following bariatric surgery

### Surgical Treatment Algorithm for Patients with Coexisting Achalasia and Morbid Obesity

**Fig. 1:**
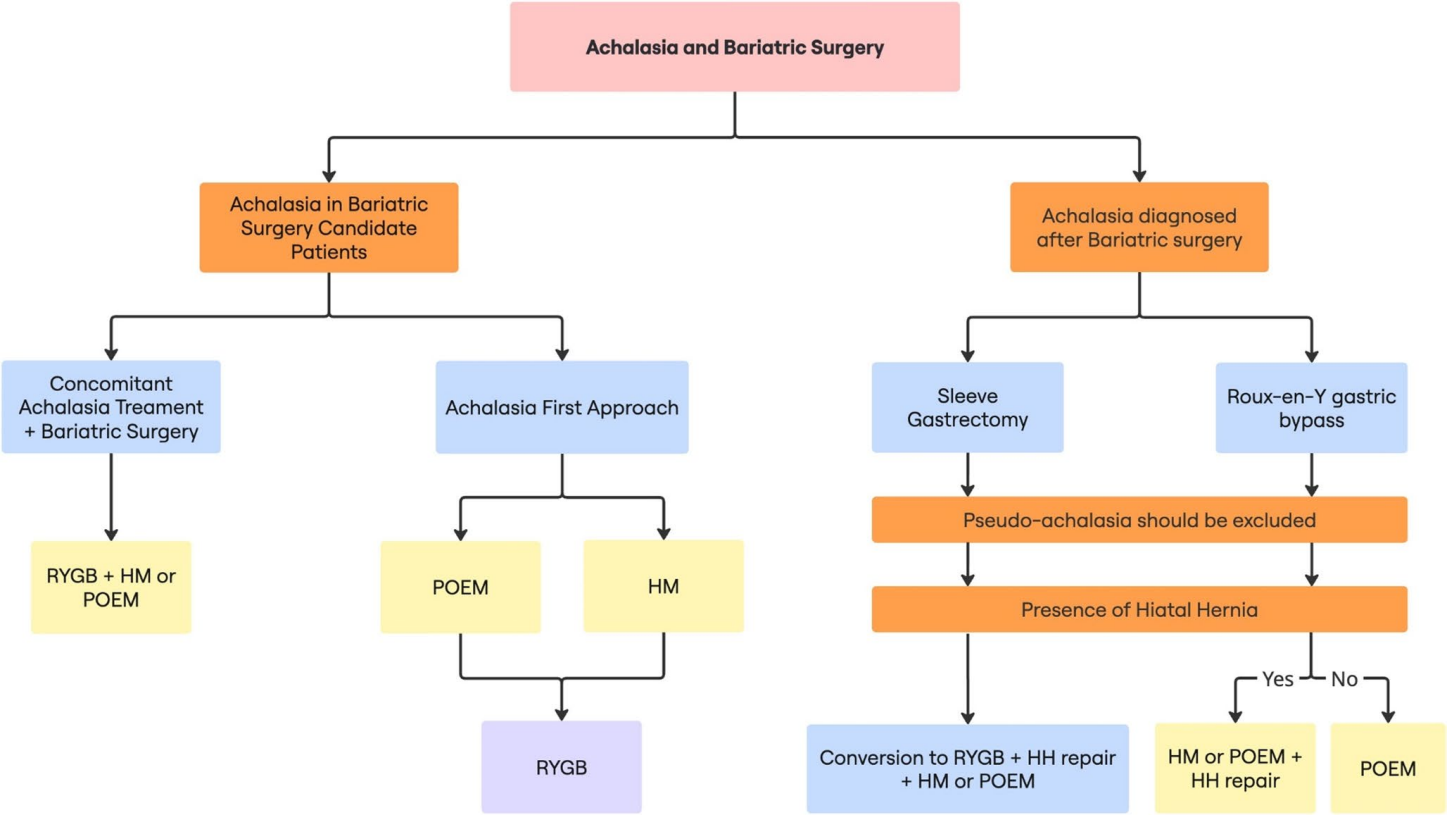
Proposed treatment algorithm for patients with achalasia and obesity. (HH = Hiatal Hernia; HM = Heller’s Myotomy; POEM = Peroral Endoscopic Myotomy; RYGB = Roux-en-Y Gastric Bypass; SG = Sleeve gastrectomy)

**Based on our institutional experience and literature review**, Fig. 1 presents our proposed preliminary treatment approach for patients with achalasia and severe obesity, stratified by the timing of achalasia diagnosis relative to bariatric surgery. The algorithm illustrates two primary pathways, achalasia diagnosed concurrently or after bariatric surgery, and outlines suggested interventions based on clinical context and prior surgical anatomy. This algorithm represents our institutional approach and requires validation in larger patient cohorts.

## Discussion

This single-institution case series adds to the limited literature on patients with coexisting achalasia and obesity who undergo treatment for both conditions. Our findings suggest that the timing of interventions whether achalasia is managed before, after, or concurrently with bariatric surgery and can influence outcomes, particularly symptom control, complication rates, and weight loss trajectories.

The interplay between obesity and EMDs and achalasia has been increasingly recognized, with studies estimating that esophageal motor abnormalities may be present to some degree in over half of patients with severe obesity [9]. Although the prevalence of achalasia in this population remains low (0.5–1%) [10], this likely reflects underdiagnosis due to symptom overlap, diagnostic challenges, and low clinical suspicion. In our cohort, patients presented with classic achalasia symptoms, dysphagia (82.4%), regurgitation (58.8%), and chest pain, mirroring prior reports in post-bariatric populations [16, 17]. Type II achalasia was the most frequent subtype, consistent with earlier studies [8]. Stratifying patients into each group revealed differences in outcomes, though our sample size limits definitive conclusions.

In our AF group, consistent with established literature, patients who underwent Botulinum toxin injection or pneumatic dilation often required further interventions, as these patients experienced symptom relapse requiring retreatment. Esophageal myotomy, whether accomplished laparoscopically or endoscopically, demonstrated better long-term symptom relief with initial clinical success rates exceeding 85–90%, supporting POEM and HM as effective treatment options for this population. Importantly, the need for additional endoscopic or surgical interventions in some patients reflects the chronic and relapsing nature of the disease rather than technical failure alone, particularly in the context of long-term follow-up and complex foregut anatomy [8, 18–20]. Subsequent bariatric surgery was feasible, though its complexity varied based on the prior achalasia procedure. RYGB after POEM was generally straightforward due to minimal adhesions, whereas RYGB following HM was more technically challenging due to prior fundoplication or hiatal scarring. Despite this, AF group achieved favorable weight loss (%TWL 20.2–33.9% at 12 months, 18–56.3% long term) and sustained symptom relief. These results support addressing achalasia prior to bariatric surgery to optimize esophageal function and reduce procedural risk. RYGB is typically favored for its superior reflux control [9, 13], though SG remains appropriate in select patients depending on GERD severity, comorbidities, and weight loss goals.

Conversely, outcomes in the BF group were more heterogeneous and recurrence is common. Since more than half of individuals with obesity and EMDs are asymptomatic, and preoperative esophageal testing is infrequently performed, it often remains unclear whether conditions such as achalasia are pre-existing or develop de novo following bariatric surgery. Our findings reinforce the importance of a low threshold for esophageal motility evaluation in bariatric candidates with dysphagia or reflux symptoms. This diagnostic uncertainty, combined with the lack of clear management guidelines, makes treatment in this population particularly challenging [3, 16, 21]. In our BF group, some patients initially underwent endoscopic therapies such as botulinum toxin injection or pneumatic dilation as temporizing measures, while others required definitive surgical intervention. Several patients experienced recurrent or refractory symptoms, with a higher incidence of re-intervention for achalasia or surgical revision likely reflecting the technical complexity of managing achalasia or EMDs after prior bariatric surgery and altered foregut anatomy. These findings are consistent with the case series by Crafts et al., but contrast with earlier case reports and small series in which most post-bariatric achalasia patients (*n* = 37) achieved symptomatic relief after interventions such as HM or POEM, with only two cases progressing to esophagectomy. Notably, the mean follow-up duration in those studies was limited (11.5 months), potentially underestimating long-term complications and treatment failures that we were able to capture in our cohort [9]. A previous study from our institution by Boules et al. identified 10 patients who developed achalasia following bariatric surgery, including eight who had undergone RYGB and two who had vertical banded gastroplasty (VBG). The median interval between bariatric surgery and the diagnosis of achalasia was six years. All patients underwent definitive surgical management: HM was performed in the RYGB group (*n* = 8), while both patients with VBG required esophagectomy due to end-stage disease. Most patients experienced symptomatic resolution following intervention.

Both POEM and HM are acceptable primary treatment modalities in this setting. POEM offers several potential advantages over HM, including the absence of abdominal incisions, shorter recovery time compared to laparoscopic surgery, and the ability to perform a longer myotomy with greater technical ease. However, HM offers the benefit of concurrent antireflux procedures and potentially lower long-term reflux risk. Across multiple small studies, POEM demonstrated high clinical success rates in treating achalasia after gastric bypass surgery and most patients experienced symptom relief, as evidenced by significant reductions in Eckardt scores, with low complication rates [22, 23]. HM can be technically challenging in patients with prior bariatric surgery due to intra-abdominal adhesions and altered anatomy, including the lack of the gastric fundus to perform a fundoplication. Nevertheless, successful outcomes with favorable symptom relief have been reported in post-bariatric surgery patients, including those with RYGB, across multiple case series [9].

Hiatal hernia status plays an important role in procedural planning for patients with a history of SG or RYGB. In RYGB patients, either POEM or HM combined with hernia repair is considered appropriate. For those with prior SG, the severity of GERD guides the management approach. Patients with severe reflux may benefit from conversion to RYGB along with myotomy and hernia repair. Slack et al. [24] reported such a strategy in a complex case involving conversion, POEM, and hernia repair.

In our BF group, GERD prevalence was higher in SG patients than in those undergoing RYGB (100% vs. 50%), and all SG patients ultimately required conversion to RYGB alongside achalasia treatment (via POEM or HM). This aligns with prior studies demonstrating that SG is associated with reduced LES pressure, impaired peristalsis, and increased reflux, whereas RYGB improves reflux with minimal impact on motility [25]. Naik et al. reported higher rates of GERD and motility disorders, including de novo achalasia, following SG and laparoscopic adjustable gastric banding (LAGB), with RYGB appearing protective [26]. Moreover, emerging data using EndoFLIP™ technology suggest higher rates of secondary esophageal motility disorders following SG and revisional bariatric procedures, with RYGB associated with comparatively lower physiological disruption [17]. In our series, EMDs were identified significantly earlier following SG compared to RYGB (6.0 vs. 19.8 years), suggesting that SG may accelerate or unmask dysmotility due to its restrictive, high-pressure design [13, 27]. Pseudoachalasia-like syndromes after bariatric surgery have also been described, often related to mechanical or anatomic factors despite normal integrated relaxation pressure (IRP) [28]. Expanding on these findings, Miller et al. reported a large retrospective study identifying manometric achalasia in 7.2% of post-bariatric patients and a newly described entity, POSED, in 5.2%. Notably, neither condition was observed in preoperative controls, and both demonstrated a time-dependent relationship, with symptoms developing up to 15 years after surgery [13]. These observations highlight the importance of careful diagnostic evaluation in bariatric patients with persistent dysphagia or reflux symptoms. Long-term follow-up and diagnostic workup, including upper endoscopy, contrast imaging, and esophageal function testing, should be considered to exclude pseudoachalasia. When pseudoachalasia cannot be clearly distinguished from true achalasia or other esophageal motility disorders, definitive achalasia-directed treatment may still be required.

Only one patient underwent concurrent treatment (HM and RYGB), achieving excellent symptom resolution and weight loss. Although this represents a single case, this outcome is encouraging and aligns with reports advocating for simultaneous surgical management in selected patients. Kaufman et al. [29] were among the first to demonstrate the feasibility and efficacy of this combined procedure, noting the added advantage of RYGB in minimizing postoperative reflux. In their report, the patient had excellent relief of dysphagia, no heartburn, and a weight loss of 100-lbs one year postoperatively. Wesp et al. [30] advocate for concurrent HM and RYGB as the preferred strategy due to its dual benefit of symptom control and weight loss. Heller myotomy appears to be the optimal procedure to combine with RYGB, as it offers superior control of postoperative reflux compared to partial fundoplication. In patients with severe obesity and achalasia who are suitable candidates for simultaneous treatment, HM and RYGB can be performed safely. This combined approach streamlines care, reduces the need for reoperation, and limits cumulative anesthesia exposure, all while providing durable long-term outcomes [31]. Nevertheless, it requires thorough multidisciplinary planning to effectively balance operative risks and procedural complexity.

This study is limited by its retrospective design, small sample size, and incomplete manometric or follow-up data in some cases. Heterogeneity in achalasia subtypes, treatments, and postoperative courses limits direct comparisons, and institutional practice patterns evolved over the study period; in addition, the retrospective design and outside referrals with incomplete prior records contributed to variability in treatment selection, particularly in earlier cases. This study is further limited by the absence of standardized symptom scores such as the Eckardt score; symptom outcomes were instead based on clinical documentation rather than validated scales, introducing some subjectivity. In addition, quality-of-life outcomes following these treatments were not captured. Despite these constraints, our findings add to the limited literature on esophageal motility disorders in the bariatric population and emphasize the need for larger, multicenter studies to inform evidence-based treatment algorithms. A key strength is the relatively long follow-up and stratification by treatment sequence, allowing for a more nuanced analysis of outcomes, including symptom control, weight loss, and complications. These results underscore the importance of standardized protocols and future research to guide surgical decision-making in this complex group.

## Conclusion

This descriptive case series highlights the clinical complexity in patients with the rare combination of achalasia and severe obesity. While limited by the small sample size, our findings suggest that treatment sequencing may influence outcomes, with achalasia therapy prior to or concurrently with bariatric surgery demonstrating more favorable results in our experience. SG was associated with potentially earlier onset of esophageal dysfunction compared to RYGB. Despite study limitations, these findings support preoperative esophageal evaluation and multidisciplinary planning. Our proposed algorithm, while preliminary, may provide initial guidance for this challenging clinical intersection. Multicenter studies are needed to validate these observations and establish standardized treatment pathways.

## Supplementary Information

Below is the link to the electronic supplementary material.

Supplementary file 1 (DOCX 26.2 KB)

## Data Availability

No datasets were generated or analysed during the current study.
