# Supplementary material for: Achalasia and Esophageal Motility Disorders in the Bariatric Surgery Population: A Retrospective Descriptive Series
Source: Obes Surg. 2026 Mar 18;36(4):1484–94. doi: 10.1007/s11695-026-08510-x (PMC13083340; doi:10.1007/s11695-026-08510-x)
Supplement: Supplementary file 1 [file 11695_2026_8510_MOESM1_ESM.docx]

| Supplement table S1. Achalasia Presenting Symptoms |
| --- |
| Symptoms | N (%) |
| Chest pain | 8 (47.1) |
| Dysphagia |  |
| To solid | 14 (82.4) |
| To liquid | 7 (41.2) |
| Regurgitation | 10 (58.8) |
| Heartburn | 9 (52.9) |

| Supplement table S2. Manometry profiles in the study cohort. |
| --- |
|  | LES Pressure (mmHg) | Esophageal peristalsis | Type of Achalasia |
| Basal | Residual (IRP) |
| Achalasia first group |
| 1 | 3.4 | 0 | Aperistalsis | II |
| 2 | N/A | N/A | N/A | I |
| 3 | 73.4 | 34.5 | Spastic | III |
| 4 | 21 | 5.9 | Aperistalsis | I |
| 5 | 60 | 23.1 | Preserved | EGJOO |
| 6 | 23.1 | 7.6 | Aperistalsis | II |
| 7 | 10.4 | 17.6 | Aperistalsis | II |
| 8 | N/A | N/A | N/A | Unknown |
| 9 | 50.6 | 19.4 | Preserved | EGJOO |
| Bariatric first group |
| 10 | 39.4 | 26.9 | Preserved | EGJOO |
| 11 | 46.8 | 35.9 | Aperistalsis | II |
| 12 | N/A | N/A | N/A | Unknown |
| 13 | 46.1 | 27.9 | Aperistalsis | II |
| 14 | 45.4 | 5.5 | Aperistalsis | II |
| 15 | 16.7 | 20.7 | Aperistalsis | II |
| 16 | 70.9 | 19.5 | Preserved | EGJOO |
| 17 | 10.9 | 6.5 | Preserved | POSED |
| LES, Lower esophageal sphincter; IRP, Integrated resting pressure; EGJOO, Esophagogastric junction outflow obstruction; POSED, Post obesity surgery esophageal dysfunction *N/A, No available data, Unknown = Achalasia diagnosis documented without subtype specification |
